# A New Multiplatform Model for Outpatient Prenatal and Postpartum Care in a Cohort of COVID-19-Affected Obstetric Patients

**DOI:** 10.3390/ijerph18105144

**Published:** 2021-05-12

**Authors:** Mar Muñoz-Chápuli Gutiérrez, Ana Durán-Vila, Javier Ruiz-Labarta, Pilar Payá-Martínez, Pilar Pintado Recarte, Julia Bujan, Miguel A. Ortega, Juan De León-Luis

**Affiliations:** 1 Department of Public and Maternal and Child Health, School of Medicine, Complutense University of Madrid, 28040 Madrid, Spain; mar.munozchapuli@salud.madrid.org (M.M.-C.G.); advila@ucm.es (A.D.-V.); franciscojavier.ruiz@salud.madrid.org (J.R.-L.); mpilar.paya@salud.org (P.P.-M.); ppintado@salud.madrid.org (P.P.R.); jaleon@ucm.es (J.D.L.-L.); 2 Department of Obstetrics and Gynecology, University Hospital Gregorio Marañón, 28009 Madrid, Spain; 3 Health Research Institute Gregorio Marañón, 28009 Madrid, Spain; 4 Department of Medicine and Medical Specialties, Faculty of Medicine and Health Sciences, University of Alcalá, Alcalá de Henares, 28801 Madrid, Spain; mjulia.bujan@uah.es; 5 Ramón y Cajal Institute of Healthcare Research (IRYCIS), 28034 Madrid, Spain; 6 Cancer Registry and Pathology Department, Hospital Universitario Principe de Asturias, 28806 Alcalá de Henares, Spain

**Keywords:** COVID-19, pregnancy, prenatal care, prenatal diagnosis, telemedicine

## Abstract

Spain was one of the epicenters of the first wave of the COVID-19 pandemic. We describe in this article the design and results of a new telephone-and-telematic multiplatform model of systematic prenatal and postpartum follow-up for COVID-19-affected women implemented in a tertiary reference hospital in Madrid. We included patients with RT-PCR-confirmed COVID-19 during pregnancy or delivery from 10 March 2020 to 15 December 2020. We had a total of 211 obstetric patients: 148 (70.1%) were tested at the onset of suspicious clinical manifestations and 62 (29.4%) were tested in the context of routine screening. Of all the patients, 60 women (28.4%) were asymptomatic and 97 (46%) presented mild symptoms. Fifty-one women (24.2%) were admitted to our hospital for specific treatment because of moderate or severe symptoms. We had no missed cases and a good adherence. The mean number of calls per patient was 2.3. We performed 55 in-person visits. We analyzed the complexity of our program over time, showing a two-wave-like pattern. One patient was identified as needing hospitalization and we did not record major morbidity. Telemedicine programs are a strong and reproducible tool to reach to pregnant population affected by COVID-19, to assess its symptoms and severity, and to record for pregnancy-related symptoms both in an outpatient regime and after discharge from hospital.

## 1. Introduction

On 31 December 2019, the World Health Organization (WHO) China Country Office was informed by Chinese authorities of 27 cases of pneumonia of unknown etiology, including 7 severe cases related geographically to the Huanan seafood market in Wuhan city, Hubei. The causal agent was identified on 7 January by Chinese authorities as SARS-CoV2 (severe acute respiratory syndrome-related coronavirus 2) responsible for the novel coronavirus disease 2019 (COVID-19). The WHO recognized the COVID-19 outbreak as a Public Health Emergency of International Concern on 30 January 2020, and on 11 March 2020, it was characterized as a pandemic [1].

The first case outside China was reported in Thailand on 13 January, and soon after, cases were reported in at least 24 countries, mostly in South and Southeast Asia but also in Europe, the USA, Canada, and the United Arab Emirates [2]. Most of the cases in January were imported, but the easy human-to-human transmission and geographic and social diverse factors led rapidly to locally transmitted clusters of COVID-19. Wuhan was locked down on 23 January, leading the way for different global approaches to limit the spread and “flatten the curve”.

The first three cases reported in Europe were in France on 24 January. On 21 February, nine countries (Belgium, Finland, France, Germany, Italy, Russia, Spain, Sweden, and the UK) reported confirmed cases. From then, a large outbreak was identified in Northern Italy and locally transmitted infection was first described outside Hubei [3].

Spain was one of the first and most affected countries in Europe and its capital, Madrid, was soon the second largest region affected by COVID-19 after Northern Italy [4]. Our hospital is a tertiary referral center for obstetric pathology located in downtown Madrid, serving 350,000 patients. On 1 March, our center confirmed the first reverse-transcriptase polymerase-chain reaction (RT-PCR) COVID-19 case, but by then, several patients had been presenting compatible symptoms. The outbreak was so rapid and fulminant that from the first week of March 2020 we began assisting several pregnant women with suggestive symptoms even before the RT-PCR testing was fully available [5]. The concern for these patients turned to a prompt worry about the safety of non-symptomatic patients and the medical staff. The Obstetrics and Gynecology section of our center understood very soon the need to implement a new model of prenatal care for COVID-19 affected patients. 

Pregnant women have been proven to be particularly vulnerable to this disease, not only because of its implications for obstetric and neonatal outcomes [6,7] but also because of the impact of isolation, economic concerns, and social and demographic factors. Psychological aspects must be always addressed during pregnancy because of the incidence of anxiety and depressive symptoms, which can be worsened by the disease but also by isolation and lockdown situations [8,9].

The purpose of this telehealth program was first to identify COVID-19 confirmed cases during pregnancy and postpartum, and then to regularly assess clinical features and to detect any alarm signs, to ensure prenatal follow-up during COVID-19 convalescence and to give patients a tool to solve any questions related to the disease during pregnancy as well as WHO guidelines on prevention and self-care at home. The new model had to be based on telemedicine and was the first prenatal follow-up telematic-based consultation implemented in our center. 

Our aim for this study was to describe this new multiplatform care model we developed during the rise of the pandemic to answer the needs of obstetric patients suffering from COVID-19 during pregnancy, labor, or postpartum, to evaluate its results as well as to assess its impact on health system organization sharing the lessons we learned from it.

## 2. Materials and Methods

We present an observational longitudinal ecological study in a hospital-based cohort of obstetric patients affected by COVID-19 confirmed disease during pregnancy or postpartum. 

Inclusion criteria for this study was confirmed RT-PCR COVID-19 cases in pregnant women previously followed-up in our center or referred to it from the beginning of the pandemic on 10 March to 15 December 2020. While we included suspected cases in our Pregnancy and COVID-19 program, we excluded them from our study since there was no possible microbiological confirmation of the disease.

**Figure 1:**
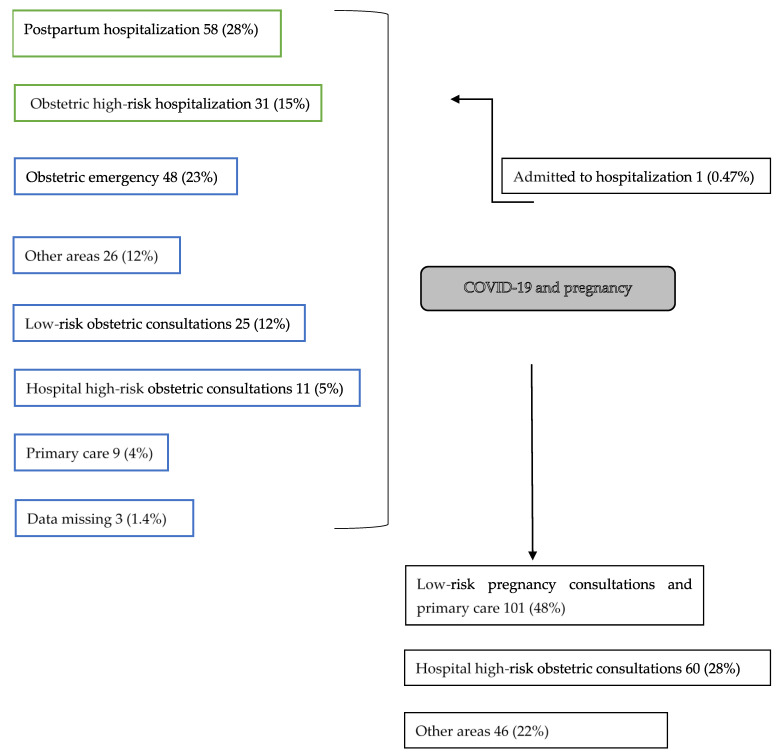
Patient flux: Recruitment from reference areas (green: inpatient, blue: outpatient) to COVID-19 and pregnancy program and discharge.

Patients were referred out of an outpatient regime from obstetric emergencies, low-risk or high-risk obstetric consultations, or primary care. There were patients referred to our Pregnancy and COVID-19 program from other hospitals in downtown Madrid. We received patients after discharge from hospital from postpartum hospitalization and obstetric high-risk hospitalization (Figure 1). We included low-risk and high-risk pregnancies. Most patients were RT-PCR tested as they presented suggestive symptoms. Later, Madrid started implementing contact testing and on 6 May we started asymptomatic screening prior to any obstetric procedure. 

Health practitioners that referred pregnant women to our Pregnancy and COVID-19 program checked contact forms, verified phone numbers, and informed the patient of the day and time of our next telephone contact. All patients included in this study agreed to participate in this project. 

We designed this outpatient model in nine days after we recorded the first RT-PCR confirmed case in a pregnant woman on 10 March. The first precept of this telehealth visit scheme was to assure full support from primary-care doctors and nurses. We planned to use HORUS Software, which is an integrative online application that allows access from any Health Service center of the community of Madrid (SERMAS) to primary and hospital health digital reports (HORUS 1.0., Horus Hardware Corp., Las Rozas de Madrid, Madrid, Spain). Primary care practitioners (nurses and medical doctors) have been regularly updating the clinical information in the same way we have updated the information collected from our telephonic and in-person follow-up. Using that tool, we were able to establish a fluent communication route from primary caregivers to hospital obstetricians. 

**Figure 2:**
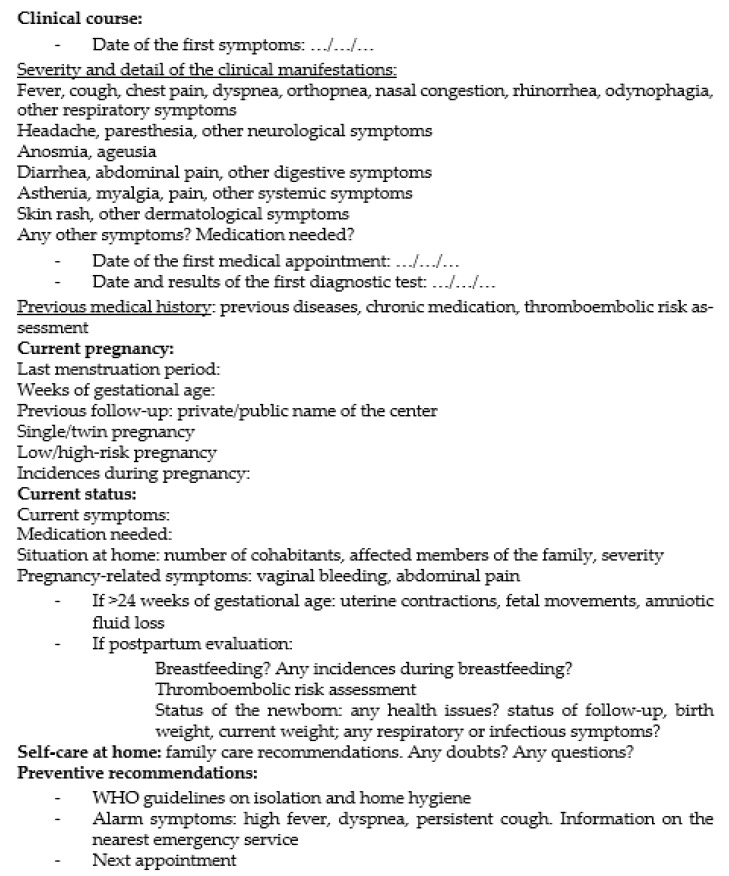
Semi-structured questionnaire.

We defined a multi-platform visit system that integrated telephone visits, telematic online communication with primary care and in-person visits when necessary. Before each telephone visit, we made sure their family practitioner had already contacted each patient and we noted their clinical evaluation. On the first telephone call, a maternal and fetal medicine specialist used a semi-structural individual questionnaire to assess the early signs of symptoms, severity and duration, prenatal follow-up until the outset of the disease, weeks of gestational age, current COVID-19 and pregnancy-related symptoms, and doubts or questions related to the process (Figure 2). We aimed to match our first telemedicine prenatal program to humane obstetric practice, which was a need COVID-19-affected pregnant patients emphasized [6,10].

Following clinical criteria, we offered weekly in-person follow-up at the hospital in the afternoon and after routine visits, with one maternal–fetal medicine expert performing routine and recommended ultrasound examinations, clinical assessment, and laboratory testing (RT-PCR when needed, serology, blood testing for genetic and diabetes screening, genital swab for group B streptococcus) according to national and international guidelines [11,12,13]. This was performed meeting every safety measure and using personal protective equipment.

We then shared the information with primary care practitioners via HORUS software and scheduled the next weekly telephone or in-person visit. We offered telephone follow-up in coordination with primary care. We established the frequency of the calls based on the severity of the symptoms according to the first questionnaire. Patients received a telephone notification before every appointment via SMS, informing them about the approximate time of the next call. In-person visits were offered when the patient needed a clinical obstetric evaluation because of pregnancy-related alarm symptoms (first or second trimester bleeding, premature uterine contractions, hypertensive symptoms) or a recommended visit was to be performed (blood test for genetic screening, morphologic or growth ultrasound exams).

When the patients were discharged from the process according to our protocol (ten days after RT-PCR positive in asymptomatic pregnant women, at least 6 days without symptoms if mild or moderate disease or RT-PCR negative or positive IgG serology after the resolution of symptoms), we arranged the next in-person visits in a low-risk or high-risk consultation based on clinical criteria and previous protocols.

We studied maternal characteristics from our population and prior obstetrical characteristics such as maternal age, body mass index (BMI), parity, previous cesarean section and previous or ongoing obstetric morbidity. We coded clinical manifestations of COVID-19 in four groups: asymptomatic when no symptoms were described during the convalescence, mild symptoms when they could be handled with unspecific treatment, moderate symptoms when at least one episode of hospitalization was needed for oxygen therapy or other specific treatment, and severe symptoms when we notified an admission on general intensive care. We assessed gestational age at diagnosis, the number of telephone visits required, the time to first contact and the time before discharge.

We used Microsoft Excel 19.0 (Microsoft Corporation, Albuquerque, New Mexico, United States)for data record and analysis. For analysis of qualitative variables, we defined clinical characteristics groups and we studied mean and variance. For quantitative variables, we estimated mean, median, and standard deviation, and we used a Student’s *t*-test for comparison of independent means when necessary. 

This study was designed in accordance with national regulations and with the principles of ethical guidelines on the Helsinki Declaration. These patients conformed a multi-purpose cohort and were included after verbal and written consent to other national and international studies approved by our Ethics Committee (Code: COVID-GESTA).

## 3. Results

We included a total of 211 pregnant patients with confirmed COVID-19 infection in our program from 10 March 2010 to 15 December 2020. We identified 148 (70.1%) pregnant women who were first tested and admitted to healthcare follow-up due to the onset of clinical manifestations, and 62 (29.4%) who were admitted after screening; one who had missing cause of testing. 

We show the flux of patients in Figure 2. Patients were mostly referred from postpartum hospitalization (*n* = 58; 28%), obstetric emergency (*n* = 48; 23%), and obstetric high-risk hospitalization (*n* = 31; 15%). 

**Figure 3:**
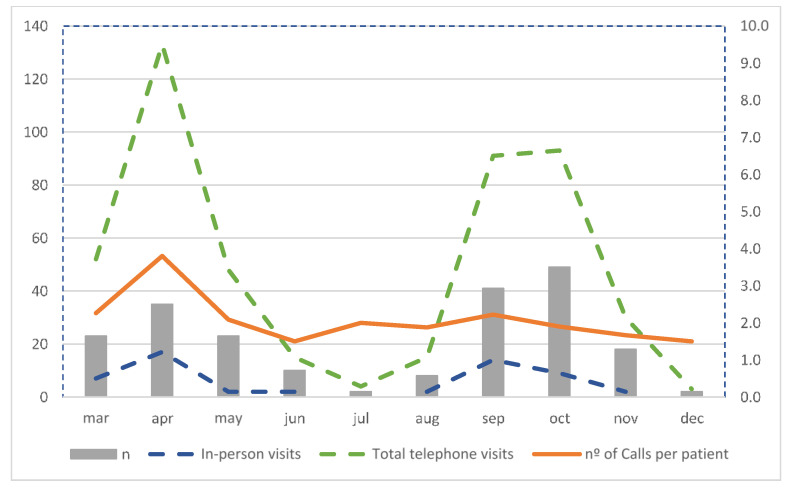
Results: in-person visits (*n*, left *y*-axis), total telephone visits (*n*, left *y*-axis), and number of calls per patient (*n*, right *y*-axis).

**Figure 4:**
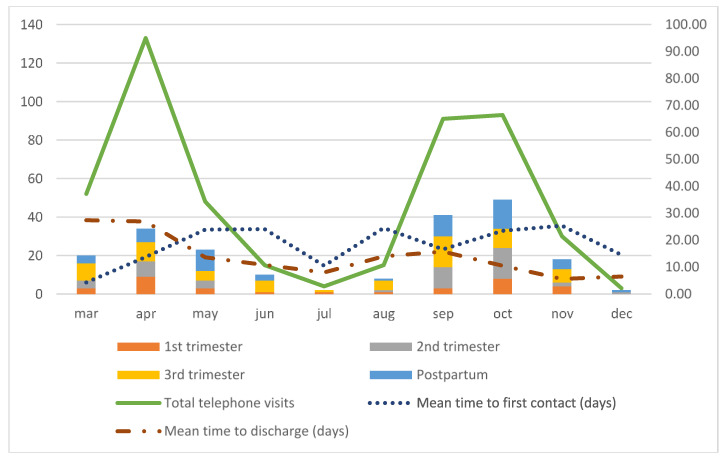
Results: new patients distributed by trimester of gestational age at the time of the diagnosis (1st, 2nd, 3rd trimesters, postpartum: *n*, left *y*-axis), total telephone visits (*n*, left *y*-axis), mean time to first contact, and mean time to discharge (days, right *y*-axis).

Table 1 presents the descriptive results of our Pregnancy and COVID-19 program. We show the number of included obstetric patients from each trimester and postpartum, the type of medical assistance, and managed times in function of time. We scheduled 40 days of consultations on a weekly basis, lowering to every two weeks from 14 July to 22 September. The estimated time for each telematic and telephone visit was 15 min. We made 484 telephone calls, which corresponds to a mean of 2.3 telephone visits per patient. We had 55 in-person visits where we performed clinical examination, high-resolution ultrasound exams, and blood tests when needed. Table 2 describes the detail of the procedures performed during in-person visits. We performed 10 first trimester blood tests and ultrasound scans, 10–20-week ultrasound scans, and 19 third trimester blood tests and ultrasound scans. The mean time to first contact was 18 days and the mean time to discharge was 16 days after the first contact. In Table 3, we evaluate the time to first contact in pregnant women for every trimester at the time of diagnosis and for postpartum patients. In the first trimester, the average time to make a first telephone call was greater (mean 28, median 18) than in the second (mean 17, median 15) and third trimesters (mean 15, median 13), and the differences between first and third trimester were significant (*p* = 0.01). Figure 3 shows the graphic evolution of these variables over time, and the distribution of total telephone visits, in-person visits, and number of calls per patient. In Figure 4 we analyze the flow of new patients from the beginning of the pandemic on 10 March to 15 December and their distribution based on the gestational age at the time of diagnosis. We also assessed the progression of the mean time to first contact and the mean time to discharge.

We describe demographic and clinical characteristics in Table 4. The mean age of our patients was 32 ± 6 years and mean body mass index was 25.6 ± 5.6 (BMI kg/m^2^ ± standard deviation). Of all the patients, 51.4% of them were primiparous, 31 (14.7%) patients had at least one previous cesarean section, and 4 (1.9%) of them had two. 

From the total number of patients, 45 (21.3%) of our patients had previous medical morbidity, from which the most frequent conditions were severe obesity (*n* = 11; 5.2%), previous oncologic disease (*n* = 8; 3.8%), hematologic conditions (*n* = 6; 2.8%), and previous thyroid disease (*n* = 6; 2.8%). Two of the women had previously confirmed gestational trophoblastic disease. The pregnancy had previously been identified as high-risk pregnancy for 38 (18%) of the patients. We had one twin pregnancy (dichorionic). In all, 45 (21.3%) of our patients had current obstetric morbidity, from which the most frequent was poor obstetric history, defined as 2 or more preterm births or late miscarriages, previous congenital defects, or stillbirths (*n* = 13; 6.2%), gestational diabetes (*n* = 10; 4.7%), intrauterine growth restriction or small for gestational age (*n* = 5; 2.3%), and preeclampsia (*n* = 3; 1.4%), among others.

In our cohort, 60 women (28.4%) were asymptomatic at diagnosis and 97 (46%) presented mild symptoms that could be managed in an outpatient scenario. Forty-one women (19.4%) were admitted during convalescence to obstetric hospitalization for specific treatment such as non-invasive oxygen support or corticosteroids and 10 (4.7%) were admitted to general hospitalization for intensive or invasive oxygen support (see Table 1). 

One patient was referred to the emergency room after telephone evaluation (0.4%), where she was then hospitalized and treated with corticosteroids and non-invasive oxygen therapy, after which she recovered and was released to continue outpatient controls. After discharge from the program, most patients were remitted to low-risk pregnancy consultations and primary care (*n* = 101; 48%) and hospital high-risk obstetric consultations (*n* = 60; 28%). Two patients interrupted voluntarily the pregnancy and two were diagnosed of morphologic malformations. No maternal or perinatal deaths were detected during follow-up.

## 4. Discussion

Our multiplatform care model scheduled 40 days of consultations on a weekly basis and made 484 calls to a total of 211 patients in a two-wave-like evolution through the pandemic. Our initial estimation of 15 min per patient and a recess for in-person evaluation in the afternoon was quickly exceeded by the incidence of COVID-19 in our population and the demands of close follow-up of these patients. We believe a more flexible program could be useful in periods of high incidence. 

We had 89 patients referred after discharge from hospital (43%) and 119 (56.4%) patients remitted from an outpatient regime from our reference areas (Figure 1). We consider that obstetricians from our service understood this program was not only a useful tool for managing mildly affected pregnant women but also a valuable tool to be used after hospitalization from moderate and severe COVID-19 manifestations. Given the results and the lack of major morbidity, we believe this program is a strong tool for COVID-19 and pregnancy follow-up after hospital discharge.

The average number of total telephone visits per patient was 2.3. Analyzing its distribution over time, we found a progressive decrease in the number of calls per patient. A similar effect was observed on the average time to discharge (Table 2, Figure 3 and Figure 4). We feel this might have been the effect of acquired knowledge from the first patients and their follow-up. Since this was a new disease and obstetricians feared its repercussions on perinatal and maternal health, we offered a more intensive follow-up to assess their progression. With time, our COVID-19 and pregnancy program settled, and we could narrow down our patient’s follow-up procedures.

We had 55 in-person visits (26% of the patients), which are shown on Table 3. We performed mostly multiple procedures in the same visit (clinical examination, ultrasound scans, and blood testing). This clearly reflects our intention to perform high-resolution visits to minimize the exposure to other patients and healthcare practitioners. 

Most of our patients presented symptoms. Only one patient from our program was remitted to the emergency room service. Many of the women shaping our cohort showed previous medical and obstetric morbidity, which did not affect our results. We plan to continue our study with the analysis of other perinatal results such as premature birth and delivery complications.

The distribution of new patients in our study mimics the two-wave incidence of COVID-19 disease. The incidence at the peak of the second wave was slightly greater that of the first wave, this might have been related to a greater availability of the RT-PCR techniques. RT-PCR was not fully available at the beginning of the pandemic, and its use was first restricted to severe patients. In our study, we analyzed the distribution of gestational age at diagnosis and the proportion of postpartum patients over time (Figure 4). The distribution was visibly homogeneous during the time of the study, but we had a mild increase of postpartum patients at the end of the first wave and in the second. Screening for RT-PCR for SARS-CoV2 started in May in our center and contact-testing public policies were implemented at the same time in our city, which could explain this fact. 

One of the main issues we found in our analysis was the time to first contact. The general mean time was 19 days, and the median was 15 days from the time of diagnosis. Since the consultations were on a weekly basis, this might have delayed our first contact. We studied the distribution of this variable in function of gestational age (Table 4) and we found differences. Comparing the time to first contact between first and third trimester we found a significant difference (*p* = 0.01). We might regard this difference as a response to an increased fear of complications of COVID-19 in third trimester pregnant women among practitioners, which is not supported by current evidence [14].

The time to make a first telephone visit did not decrease significantly over time and though we found a greater length of time detected in patients referred from an inpatient regime, after hospital discharge, compared to outpatient regime, we did not find statistical significance. This variable obeys as far as we know to several factors that were out of our control, the main one being administrative issues in the citation system in our center and in the communication between primary and hospital care. We are currently working on this issue and our aim is to decrease the length of time to first contact in the following weeks.

We found other limitations to our study. Our COVID-19 and Pregnancy program was mainly performed by a single maternal and fetal medicine specialist. This was appreciated by the women comprising this program because the follow-up could be personalized and close, but it led to a greater workload. At the acme of maximum incidence this might increase the risk of burnout syndrome; thus, we recommend creating small workgroups to reproduce this program. Because of the healthcare pressure we could not offer the patients a way of contacting the program outside of the scheduled appointments. A two-way communication system would be recommended for further multiplatform models. We could not compare our results to a control group as we developed our COVID-19 and Pregnancy program to give a response to every COVID-19 affected patient and this limited the interpretation of our results.

One of the main strengths of our program was the constant dialogue with primary care. This helped us sort the issue of time to first contact, because when we first contacted the patients, primary care had already started their follow-up program. We could record via the HORUS application retrospectively the progression of the symptoms and thus adapt our obstetric follow-up. The first difficulty we perceived when designing this telehealth model relied first on the heterogeneity of the software used in our region. We know this has been a problem in the onset of new prototypes of telemedicine programs [15,16] all around Europe. COVID-19 has shown the urgent need to universalize the access to health information ensuring data protection. Telehealth systems will naturally thrive when this happens, for they are a strong, reproducible and useful tool, also in obstetrics and perinatal medicine [17,18], allowing the safety of health workers and other pregnant patients without compromising obstetric recommended procedures [19] and offering a secure, close and overall satisfying medical attention [20].

Other programs of telemedicine have been described to address the issue of obstetric follow-up in a pandemic context. Trostle et al. [21] described a telephone-based program of outpatient follow-up and classification of COVID-19 risk symptoms. Clinical manifestations were similar to those found in our cohort, with a majority of mildly symptomatic patients. Its strength compared to our program was the rapid response of their “COVID-19 list” model since many practitioners had access to it. However, they included a large proportion of non-confirmed cases. We presented a much wider cohort of patients and we studied the progression of our response over time, but more importantly, we believe our combined telephone/in-person program and coordination with primary care has given patients a much closer and more humane approach. We intend to study its impact on mental health and the experience of childbearing and postpartum. 

A similar program based on an email list was also performed in the United States [22,23] with a mixed telephone and video system. In both cases this was not the first telemedicine program in obstetric care; therefore, the rate of no-visits was relevant. In our case we had no missed calls and adherence to the program was satisfactory. We believe primary care liaison played a capital role in this. The simplicity of our joined telephone and telematic approach helped us iron out the inequalities and guarantee the access to prenatal follow-up.

To our knowledge, this study presents one of the largest cohorts of COVID-19-affected pregnant women followed-up by a novel telemedicine program and the first study to assess its evolution during different waves of the pandemic.

## 5. Conclusions

We presented a new multiplatform telehealth model, our COVID-19 and Pregnancy program, with a combination of computer-based software and telephone visits, created from the urge of the pandemic, to guarantee prenatal assessment of COVID-19-affected pregnant women. This has been the first telematic program implemented in our obstetric center. The strengths of this project are the connection with primary care, which played a capital role in the follow-up of these patients and the rapid creation of a network of caregivers who introduced the patients to the program. This COVID-19 and pregnancy program demonstrated its effectiveness in following-up mildly affected pregnant women and ensuring home care after hospital discharge.

## Figures and Tables

**Table 1:** Descriptive results of Pregnancy and COVID-19 program: obstetric patients (*n*; number) from each trimester and postpartum, type of medical assistance, and managed times (days) in function of time (months of study).

Month	*n*	1st Trimester (*n* (%))	2nd Trimester (*n* (%))	3rd Trimester (*n* (%))	Postpartum (*n* (%))	In-Person Visits (*n* (%))	Total Telephone Visits (*n*)	Number of Telephone Visits per Patient (*n*)	Mean Time to First Contact (days)	Mean Time to Discharge (days)
Mar.	23	3	4	9	4	7	52	2.3	4.3	27.4
Apr.	35	9	8	10	7	17	133	3.8	13.7	26.9
May	23	3	4	5	11	2	48	2.1	23.9	13.6
Jun.	10	1	-	6	3	2	15	1.5	24.1	10.8
Jul.	2	1	-	1	-	-	4	2.0	10.5	8.0
Aug.	8	1	1	5	1	2	15	1.9	24.5	14.0
Sep.	41	3	11	16	11	14	91	2.2	16.6	15.7
Oct.	49	8	16	10	15	9	93	1.9	23.5	10.5
Nov.	18	4	2	7	5	2	30	1.7	25.3	5.6
Dec.	2	-	1	-	1	-	3	1.5	14.5	6.5
Total	211	33 (15.6)	47 (22.2)	69 (32.7)	58 (27.5)	55 (26.1)	484	2.3	18.7	16.0

**Table 2:** Detail of procedures performed during in-person visits.

1st Tr Blood Test	-
12-w ultrasound	1
16-w early ultrasound scan	1
Blood test 1ºT and 12-w ultrasound	10
2nd Tr blood test	-
20-w ultrasound	10
2nd Tr blood test and 20-w ultrasound	1
3rd Tr blood test	-
32–34 w ultrasound	8
3rd Tr blood test and 32–34-w ultrasound scan	19
Growth ultrasound scan	2
Other	3
Total	55

**Table 3:** Mean time to first contact for pregnant and postpartum patients (days).

	Mean Time to First Contact	Median Time to First Contact	Standard Deviation	Student’s *t*-Test (*p*)
1Trimester	28	18	31	2.2 (0.01) **
2Trimester	17	15	13	
3Trimester	15	13	11	
Total pregnant	19	15	18	
Postpartum	19	15	14	
Inpatients	20	16	13	
Outpatiens	18	14	13	
Total	19	15	17	

** T Student’s test for comparison of first to third trimester mean time to first contact.

**Table 4:** Demographic and clinical characteristics.

Maternal Characteristics	Measure (Mean)	Range (Variance)
Age	32.2 years	6
BMI	25.6 Kg/m^2^	5.6
Obstetrical characteristics	*n*	%
Primipara	108	51.4
Previous cesarean section	*n*	%
One	27	12.9
Two	4	1.9
Clinical manifestations	*n*	%
Asymptomatic	60	28.4
Mild symptoms	97	46
Moderate Symptoms	41	19.4
Severe symptoms	10	4.7

## Data Availability

The data used to support the findings of the present study are available from the corresponding author upon request.
